# SMN post-translational modifications in spinal muscular atrophy

**DOI:** 10.3389/fncel.2023.1092488

**Published:** 2023-02-17

**Authors:** Giulietta M. Riboldi, Irene Faravelli, Paola Rinchetti, Francesco Lotti

**Affiliations:** Center for Motor Neuron Biology and Diseases, Departments of Pathology & Cell Biology, and Neurology, Columbia University Irving Medical Center, New York, NY, United States

**Keywords:** survival motor neuron, spinal muscular atrophy, post-translational modifications, mRNA splicing, ribonucleoproteins

## Abstract

Since its first identification as the gene responsible for spinal muscular atrophy (SMA), the range of survival motor neuron (SMN) protein functions has increasingly expanded. This multimeric complex plays a crucial role in a variety of RNA processing pathways. While its most characterized function is in the biogenesis of ribonucleoproteins, several studies have highlighted the SMN complex as an important contributor to mRNA trafficking and translation, axonal transport, endocytosis, and mitochondria metabolism. All these multiple functions need to be selectively and finely modulated to maintain cellular homeostasis. SMN has distinct functional domains that play a crucial role in complex stability, function, and subcellular distribution. Many different processes were reported as modulators of the SMN complex activities, although their contribution to SMN biology still needs to be elucidated. Recent evidence has identified post-translational modifications (PTMs) as a way to regulate the pleiotropic functions of the SMN complex. These modifications include phosphorylation, methylation, ubiquitination, acetylation, sumoylation, and many other types. PTMs can broaden the range of protein functions by binding chemical moieties to specific amino acids, thus modulating several cellular processes. Here, we provide an overview of the main PTMs involved in the regulation of the SMN complex with a major focus on the functions that have been linked to SMA pathogenesis.

## Introduction

Since its identification in 1995 as the disease-causing gene product of spinal muscular atrophy (SMA; Lefebvre et al., 1995), the landscape of survival motor neuron (SMN) protein functions has expanded tremendously. SMN is an evolutionary conserved and ubiquitously expressed protein that localizes to both the cytoplasm and the nucleus, where it concentrates in nuclear bodies termed Gemini of Cajal bodies (Gems; Liu and Dreyfuss, 1996). *SMN1* gene mutations or deletions are responsible for SMA, which represents the leading cause of death for a genetic disorder in infants (Lefebvre et al., 1995).

Humans hold on the same chromosome a paralogous gene, *SMN2*, that differs from *SMN1* for a single nucleotide leading to the exclusion of exon 7 in the transcript and to the production of a truncated SMN protein (also known as SMNΔ7), rapidly degraded. Remarkably, the number of copies of *SMN2* inversely correlates with the severity of the disease phenotype in SMA. However, *SMN2* copies can be structurally different between SMA patients and other genetic modifiers of the disease have been identified that add complexity to the clinical picture and need to be taken into account in the context of prenatal counseling (Wadman et al., 2020).

SMA is characterized by progressive degeneration of the spinal motor neurons, leading to paralysis and respiratory failure. In addition, studies in SMA animal models have identified multiple perturbations in the motor circuit that include dysfunction and loss of neuromuscular junctions (NMJs) and central proprioceptive sensory synapses onto motor neurons (Mentis et al., 2011; Tisdale and Pellizzoni, 2015).

SMN exerts its major functions as part of a multi-subunit complex comprised of eight additional core components (Gemins2–8 and Unrip). Although the SMN complex is likely a multifunctional machinery involved in several aspects of RNA metabolism (Burghes and Beattie, 2009; Li et al., 2014; Donlin-Asp et al., 2016), its best-characterized function is in the assembly of small nuclear ribonucleoproteins (snRNPs) of the major (U2-dependent) and minor (U12-dependent) spliceosomes (Meister et al., 2001; Pellizzoni et al., 2002). In addition to spliceosomal snRNPs, the SMN complex is required for the assembly of a variant core comprising Sm and Sm-like (LSm10 and LSm11) proteins on the U7 snRNA (Pillai et al., 2003; Tisdale et al., 2013). U7 snRNP functions in the unique 3’-end processing of replication-dependent histone mRNAs that comprise the most abundant class of intron-less and non-polyadenylated transcripts in metazoans (Marzluff et al., 2008). Accordingly, SMN deficiency impairs U7 biogenesis, leading to histone mRNA processing deficits in SMA (Tisdale et al., 2013).

Increasing evidence link the function of the SMN complex in snRNP biogenesis with the etiology of SMA (Chari et al., 2009; Li et al., 2014). Defects in snRNP assembly correlate with disease severity and lead to a reduction in the steady-state levels of snRNPs in mouse models of SMA (Gabanella et al., 2007; Zhang et al., 2008). In addition, the injection of purified snRNPs in SMN-deficient embryos was shown to suppress motor axon outgrowth deficits in a zebrafish model of SMA (Winkler et al., 2005), while selective enhancement of the U12 splicing by adeno-associated virus-mediated overexpression of minor snRNAs prevented the loss of proprioceptive synapses onto motor neurons in SMA mice (Osman et al., 2020). The predicted outcome of reduced snRNP assembly is an alteration in splicing due to reduced snRNP levels. Importantly, several studies identified SMN-dependent splicing events that are essential for motor neuron function *in vivo* and linked defective splicing of these genes to sensory-motor dysfunction and to the death of motor neurons in SMA models (Lotti et al., 2012; Simon et al., 2017, 2019; Van Alstyne et al., 2018; Osman et al., 2020; Tisdale et al., 2022). These findings highlighted the impairment of SMN function in snRNP assembly as one mechanism contributing to SMA pathology.

In addition to its role in snRNP biogenesis, SMN has been reported to be involved in a number of other RNA-mediated processes. A large body of literature documented the interaction of SMN with several RNA-binding proteins (RBPs) involved in many aspects of RNA metabolism, including splicing, transport, stability, and translation of mRNAs (Li et al., 2014; Donlin-Asp et al., 2016). The fact that SMN interacts with several RBPs that are not involved in snRNP biogenesis, combined with the observation that SMN can localize to mobile granules in axons, suggested a role for SMN in axonal mRNA metabolism. Consistent with this, SMN colocalizes in neuronal granules with hnRNP R, FMRP, HuD, KSRP, and IMP1 (Rossoll et al., 2002, 2003; Piazzon et al., 2008; Fallini et al., 2011, 2014). In addition, granules containing ectopically expressed fluorescently-tagged SMN exhibit rapid, bidirectional movement in the axons of cultured neurons (Zhang et al., 2003, 2006; Fallini et al., 2010). Moreover, an alternatively spliced form of SMN (axonal-SMN or a-SMN) has been found in the axons of motor neurons and its upregulation resulted in axonal-like development in non-neuronal cells and accelerated motor neuron axonogenesis in a time-dependent manner (Setola et al., 2007). In agreement with the role of SMN in mRNP transport, β-actin and other mRNAs have reduced axonal localization upon SMN deficiency (Rossoll et al., 2003; Akten et al., 2011). Defects in mRNA localization are accompanied by a similar decrease in axonal levels of RBPs such as HuD and IMP1 as well as by reduced levels of polyadenylated mRNA-containing granules (Fallini et al., 2011, 2014; Rage et al., 2013). Thus, akin to its role in snRNP assembly, SMN may function to directly assemble mRNP complexes by increasing the affinity of RBPs for their target mRNAs (Donlin-Asp et al., 2016, 2017). These cellular processes are tightly linked to mRNA translation and recent evidence has pointed to a direct role for SMN in its regulation. Consistent with this, SMN associates with and affects the subcellular distribution of components of the translation machinery which have an impaired translational rate in SMN-depleted cells (Gabanella et al., 2016, 2020). In addition, SMN has been reported to associate with ribosomes (Sanchez et al., 2013) with SMN deficiency resulting in a reduced number of ribosomes associated with polysomes and consequent impairment of translation-related transcripts in SMA mice (Bernabò et al., 2017). Remarkably, the population of ribosomes interacting with SMN are associated with a specific subset of mRNAs that form functionally related clusters suggesting that SMN can act as a master modulator of ribosome heterogeneity on a subset of disease-relevant mRNAs (Lauria et al., 2020).

**Figure 1:**
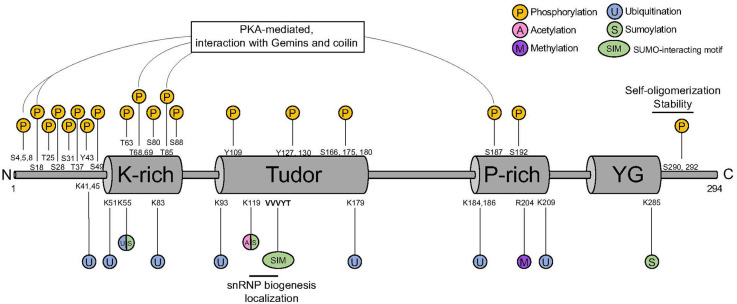
SMN protein PTM sites. SMN harbors a K-rich domain, a Tudor domain, a P-rich domain, and a YG domain that are binding sites for different PTM. P, phosphorylation; U, ubiquitination; S, sumoylation; A, acetylation; M, methylation; SIM, SUMO-interacting motif; SMN, survival motor neuron; PTM, post-translational modification.

Full-length SMN contains highly conserved functional domains that are important for its stability and functions (Figure 1). At its N-terminal, SMN harbors an interaction motif for binding to Gemin2, important for stabilizing SMN self-oligomerization and complex formation (Feng et al., 2005; Ogawa et al., 2007; Zhang et al., 2011; Grimm et al., 2013). At its center, SMN contains a Tudor domain that is involved in the interaction with di-methylated proteins, including Sm proteins (Bühler et al., 1999; Selenko et al., 2001). At its C-terminal SMN harbors a YG box required for its self-oligomerization (Martin et al., 2012) and for interaction with other Gemins (Zhang et al., 2011). The multifunctional nature of the SMN complex requires its interaction with many different protein partners, but how the specificity of these protein-protein interactions is achieved is not known. Many different mechanisms were reported as modifiers of SMN, although the full picture still needs to be elucidated. Post-translational modifications (PTMs) have been shown to play a major role in regulating the pleiotropic functions of the complex. Here, we provide an overview of the PTMs involved in the regulation of the SMN complex functions with a focus on those that have been linked to SMA etiology.

## Phosphorylation

**Figure 2:**
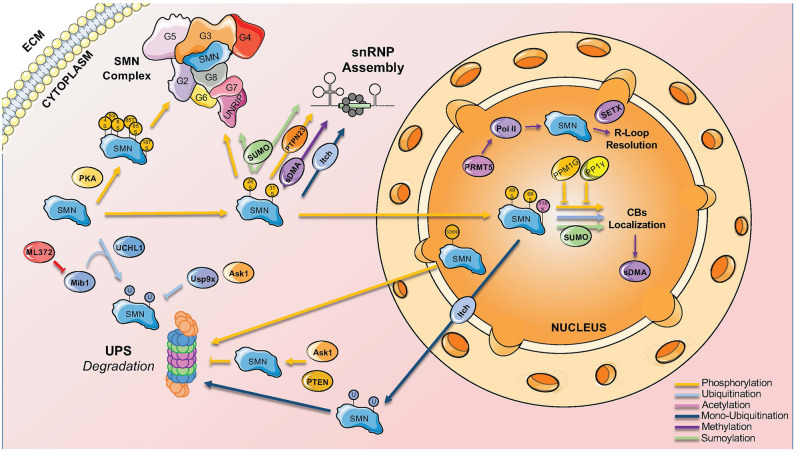
Main PTMs involved in SMN complex functions and localization. SMN complex functions and nucleocytoplasmic location are finely regulated by different PTMs that bind to specific sites on the complex.

Phosphorylation is one of the first modification type identified in SMN (La Bella et al., 2004). Out of the 50 putative phosphorylation sites present in SMN, 26 of them have been identified by mass spectrometry (Husedzinovic et al., 2014; Detering et al., 2022; Table 1). The phosphorylation sites span across the entire protein, with a mild enrichment in the N-terminus (Figure 1). In addition to SMN, a myriad of phosphorylation sites has also been identified in other members of the SMN complex, suggesting a major impact of this PTMs on the function of the complex (Figure 2).

**Table 1:** Site of SMN PTMs and their functions.

**PTM**	**Site**	**Effect**	**Reference**
***Phosphorylation***	S28, S31	snRNP assembly	Grimmler et al. (2005)
	S49, S63	Condensation of SMN protein in CBs	Schilling et al. (2021)
	S290, S292	Self-oligomerization, protein stability	Rademacher et al. (2020)
	Y109, Y127, Y130	Localization in CBs	Husedzinovic et al. (2014)
	S4, S5, S8, S18, S25, S31, T37, Y43, S49, T68, T69, S80, T85, S88, S166, S175, S180, S187, S192	PKA-mediated, interaction with Gemins and coilin	Husedzinovic et al. (2014) and Detering et al. (2022)
***Methylation***	R204	Modulation of snRNP biogenesis	Guo et al. (2014)
***Ubiquitination***	U41, U45, K51, K55, K83, K179, K184, K186, K209	SMN degradation through UPS	Danielsen et al. (2011), Kim et al. (2011), Wagner et al. (2011), Han et al. (2012), Povlsen et al. (2012), and Akimov et al. (2018)
***Acetylation***	K119	Interaction with proteins of the SMN complex (i.e. Sm, coilin); increased SMN stability (SMN turnover reduction)	Lafarga et al. (2018)
***SUMOylation***	K55, K119, VVVYT, K285	Spliceosomal snRNPs and SMN complex assembly; localization in CBs; sensory-motor circuit integrity	Tapia et al. (2014) and Riboldi et al. (2021)

The table summarizes the known sites of PTM (phosphorylation, methylation, acetylation, SUMOylation) of SMN and their effect on SMN function and biological processes. UPS, ubiquitin proteasome system; CBs, Cajal bodies.

Phosphorylation was initially described to modulate SMN complex function, stability, and subcellular distribution. In particular, the phosphorylation of serines 28 and 31 has been functionally linked to snRNP assembly activity of the SMN complex (Grimmler et al., 2005). In addition, more active SMN complex has been identified in the cytoplasm and linked to a higher level of phosphorylation (Grimmler et al., 2005). The finding that phosphatase treatment mobilizes cytoplasmic SMN to the nucleus suggested that the phosphorylation status of SMN acts also on its subcellular distribution. Indeed, non-phosphorylatable SMN mutants showed significantly less efficient accumulation in the nucleus compared to the corresponding wild-type protein, while the phospho-mimetic mutants frequently displayed cytoplasmic accumulations (Husedzinovic et al., 2014). This suggests that some of the identified sites regulate specific functions of SMN or the exchange of SMN between the nucleus and the cytoplasm and raised the hypothesis that dephosphorylation would primarily act on the nuclear SMN complex. However, this simple model of an asymmetric distribution of phosphorylated forms of the SMN complex has been recently challenged by observations that nuclear phosphorylation of SMN on serines 49 and 63 are crucial for the condensation of the SMN protein in Cajal bodies (CBs; Schilling et al., 2021).

Phosphorylation has also been reported to influence SMN stability by modulating its capability to self-oligomerize and to be incorporated into high molecular weight complexes. Both the Tudor domain at the SMN N-terminus and the YG-box at its C-terminus contribute to SMN self-oligomerization. However, only phosphorylation of the C-terminal affected SMN self-oligomerization capability while phosphorylation of the N-terminus showed no impact (Rademacher et al., 2020). In addition, the SMN phospho-mimetic S290D revealed a remarkably reduced protein stability and a consequent reduction in the number of SMN-containing CBs (Rademacher et al., 2020). Furthermore, SMN phosphorylation by phosphokinase-A (PKA) at non-canonical sites (Figure 1) promotes its stability by facilitating SMN interaction with Gemins and its incorporation into high molecular weight complexes (Burnett et al., 2009; Wu et al., 2011). SMN protein stability is determined also by its binding with phosphatase and tensin homolog (PTEN; Rademacher et al., 2020) and with ASK1, a serine-threonine protein kinase involved in neuronal differentiation and mitogen-activated protein kinase (MAPK) pathway (Takeda et al., 2000; Matsuzawa et al., 2008; Kwon et al., 2011). Interaction with Gemin5 also potentiates ASK1 function (Kim et al., 2007). It has been proposed that ASK1 could act as a novel binding partner of SMN and controls its stability through complex formation (Kwon et al., 2011). Even though SMN is phosphorylated by ASK1 *in vitro*, the kinase activity of ASK1 seems to not be involved in its ability to stabilize SMN, as ectopic expression of ASK1 or of a kinase-inactive mutant equally increased SMN protein levels (Kwon et al., 2011).

Although there is substantial knowledge on the kinases targeting SMN, less is known about the involved phosphatases. The co-purification of Gemin3 and Gemin4 with the Serine-Threonine phosphatase PPP4 provided the first evidence for phosphatases interacting with the SMN complex (Carnegie et al., 2003). Overexpression of the regulatory subunits of PPP4 stimulated the accumulation of SMN in CBs suggesting a positive influence on SMN complex activity in snRNP assembly (Figure 2). Two other phosphatases were identified as interaction partners of SMN, the nuclear protein phosphatase magnesium-dependent 1 gamma (PPM1G) and the protein phosphatase 1 γ (PP1γ). In co-immunoprecipitation assays, Gemin8 could be purified together with PP1γ suggesting an indirect interaction with SMN. PP1γ mediates the accumulation of SMN in CBs of human cells in culture. Phosphorylated forms of SMN itself increase after the knockdown of PP1γ (Renvoisé et al., 2012). PPM1G knockdown leads to hyperphosphorylation of both SMN and Gemin3, which goes along with a loss of the accumulation of SMN in CBs (Petri et al., 2007). The identification of PPM1G and PP1γ as modulators of SMN complex function has been confirmed in a comprehensive screen for human phosphatases interacting with the SMN complex (Husedzinovic et al., 2015). This screen highlighted also PTPN23 as a novel regulator of SMN complex functions (Husedzinovic et al., 2015). PTPN23 is catalytically inactive as it carries a conserved alanine in a position that usually requires a serine for catalytic activity. The binding of PTPN23 to SMN may therefore stabilize its tyrosine-phosphorylation state but not dephosphorylate the protein (Husedzinovic et al., 2015). Indeed, the loss of tyrosine-phosphorylations within the Tudor domain of human SMN (Y109, Y127, Y130) abolishes SMN accumulation in CBs (Husedzinovic et al., 2014), suggesting that tyrosine-phosphorylation and its maintenance by PTPN23 are required for the nucleocytoplasmic trafficking of SMN and for ongoing snRNP assembly in the cytoplasm (Figure 2).

## Methylation

Methylation and demethylation are also important in regulating SMN complex activity. SMN is a Tudor protein and this domain is known to interact with methylated arginine (R) or lysine (K) residues (Huyen et al., 2004; Botuyan et al., 2006; Huang et al., 2006; Chen et al., 2011). Mono- or di-methylation of arginine is particularly relevant in this context (Gary and Clarke, 1998). The process is mediated by six different arginine methyltransferases (PRMT1-PRMT6). In glycine- and arginine-rich regions (GARs), arginines can be dimethylated symmetrically (sDMA) or asymmetrically (aDMA; Gary and Clarke, 1998). sDMA is driven exclusively by PRMT5, which complexes in the cytoplasm with pICln and MEP50 and recruits Sm proteins *via* the pICln subunit (Friesen et al., 2001; Meister et al., 2001). sDMA is crucial for modulating snRNP biogenesis by targeting the C-terminal tail domains of SmB, D1, and D3, which is believed to facilitate their transfer onto the SMN complex (Brahms et al., 2001; Friesen et al., 2001; Meister et al., 2001). Accordingly, inhibition of methylation causes the loss of methylated SmB and abolishes the formation of CBs (Figure 2; Boisvert et al., 2002).

Arginine methylation is also important for the process of condensation and for the formation of membrane-less organelles (MLOs) such as CBs and Gems (Courchaine et al., 2021). The Tudor domain of SMN is critical in this context by binding DMA-containing proteins and providing the specificity required for the formation of endogenous MLOs (Courchaine et al., 2021). The implication of this observation is that biomolecular condensation can be driven by specific interactions of each Tudor domain with its DMA ligands (Courchaine et al., 2021). In line with these observations, an early report showed that sDMA substrates are enriched in CBs and cells derived from SMA patients show a granular pattern of sDMA in the nucleus (Boisvert et al., 2002).

In addition to its role in snRNP biogenesis, symmetrical dimethylation by PRMT5 of the carboxyl-terminal domain of Polymerase II in arginine R1810 has been reported to facilitate the recruitment of SMN and senataxin to form a complex responsible for R-loops resolution during transcriptional termination (Figure 2; Zhao et al., 2016).

Finally, in a proteomic study of arginine-methylated proteins in the mouse brain, SMN has been reported to contain a monomethyl group at its R204 residue (Figure 1 and Table 1, Guo et al., 2014). Although the functional implication of this SMN’s arginine modification has not been investigated yet, it can be crucial for SMN to facilitate interactions with its numerous protein partners.

## Ubiquitination

Degradation of both full-length SMN and its truncated form lacking exon 7 (SMNΔ7) is achieved by the ubiquitin-proteasome system (UPS; Chang et al., 2004; Burnett et al., 2009). Several ubiquitination sites have been described for SMN, with at least 10 of them confirmed to be ubiquitylated by proteomics (Figure 1 and Table 1, Danielsen et al., 2011; Kim et al., 2011; Wagner et al., 2011; Han et al., 2012; Povlsen et al., 2012; Akimov et al., 2018). Early studies showed that SMN levels are increased upon inhibition of the proteasome (Chang et al., 2004; Burnett et al., 2009). Consistent with this, E3 ubiquitin ligases, deubiquitinases, and other factors influencing SMN stability through ubiquitination have been identified (Figure 2).

The first mediator of SMN ubiquitination identified is the Ubiquitin carboxy-terminal hydrolase L1 (UCHL1) protein, also known as PGP 9.5. This is a very abundant neuron-specific protein that has been shown to interact with SMN in P19 and NSC34 cells (Hsu et al., 2010). Also, its expression is increased in SMA patient fibroblasts and in mouse models, while it is significantly reduced in induced pluripotent stem cells (iPSC)-derived motor neurons from SMA patients (Hsu et al., 2010; Fuller et al., 2016). In SMA fibroblasts and in animal models, UCHL1 increase is likely a compensatory response to the reduction of Ubiquitin-like modifier activating enzyme 1 (Uba1), indicative of an attempt to restore aberrant ubiquitination caused by low SMN levels (Powis et al., 2014). Consistent with this, pharmacological inhibition of UCHL1 has been shown to exacerbate rather than improve SMA symptoms in a mouse model of the disease (Powis et al., 2014).

A second E3 ubiquitin ligase acting on SMN is the multi-domain protein mind bomb 1 (Mib1). Mib1 is involved in a broad spectrum of functions, ranging from modulation of apoptosis, tyrosine kinase receptors ubiquitination, and promotion of post-mitotic neurons differentiation through the Delta-Notch signaling pathway (Jin et al., 2002; Choe et al., 2007; Berndt et al., 2011). One study reported interaction between SMN and Mib1 with overexpression of the latter increasing ubiquitination and degradation of SMN in cultured cells (Kwon et al., 2013). Importantly, the binding with SMNΔ7 was stronger compared to full-length SMN, probably because of inefficient oligomerization of the truncated protein and impaired interaction with E3 ubiquitin ligases (Zhang et al., 2003; Kwon et al., 2013). This could contribute to the higher instability of the SMNΔ7 protein. Remarkably, Mib1 knockdown caused an increase in SMN levels in cultured cells and improved a neuromuscular defect in *Caenorhabditis elegans* deficient in SMN. Consistent with the physiological role of Mib1 in modulating SMN, pharmacological inhibition of Mib1 was shown to partially rescue the disease phenotype in a mouse model of SMA (Abera et al., 2016).

While UCHL1 and Mib1 are the ubiquitin ligases that mainly drive the degradation of SMN, Itch is a ubiquitin ligase that modulates SMN localization (Han et al., 2016). Specifically, Itch interacts and monoubiquitinates SMN promoting its nuclear export. In addition, cell expressing a mutant of SMN deficient for ubiquitination present impaired CBs and coilin/Sm co-localization, suggesting that mislocalization of SMN disrupts CB integrity and likely impairs snRNP maturation (Han et al., 2016). However, the conclusions of this work are hard to interpret since all 22 lysines of SMN are mutated to arginines, therefore this mutant does not discriminate between the loss of ubiquitination and other lysine PTMs acting on SMN (see below).

Ubiquitin on SMN can also be removed by deubiquitinases (DUBs). USP9X is a deubiquitinating protein that interacts with the SMN complex (Han et al., 2012). It holds a ubiquitin-like domain and a ubiquitin C-terminal hydrolase domain and acts on ubiquitin lysine residues in mono- and poly-ubiquitinated proteins (Nijman et al., 2005). ASK1, which directly interacts with SMN, is one of the USP9X substrates and can recruit USP9X itself, thus inhibiting the poly-ubiquitination of SMN (Nagai et al., 2009; Kwon et al., 2011). Knockdown of USP9X in cells induces alterations of SMN stability, protein level, and function, with no modification on SMN mRNA levels (Han et al., 2012). Similar results are reported for Gemin8, while Gemin2 and 3 decrease only in overall proteins level, with no modification in terms of increased degradation (Han et al., 2012). While full-length SMN is mostly monoubiquitinated, SMNΔ7 is mostly polyubiquitinated (Boutet et al., 2007). This could explain the reason why SMNΔ7 is less stabilized by USP9X and its degradation by the UPS is more prominent. Moreover, SMNΔ7 has a prominent nuclear localization, while USP9X is mostly cytoplasmic (Zhang et al., 2003).

In addition to the UPS, a role for autophagy in regulating SMN protein levels has been proposed, however it is not clear whether this pathway is inhibited (Custer and Androphy, 2014; Periyakaruppiah et al., 2016; Rodriguez-Muela et al., 2018) or stimulated (Piras et al., 2017; Gonçalves et al., 2018) by SMN deficiency. Mechanistically, it has been reported that SMN degradation is mediated by its ubiquitination and interaction with the autophagy receptor p62 (Periyakaruppiah et al., 2016; Rodriguez-Muela et al., 2018). Although depletion of p62 levels partially rescues motor neuron death *in vitro* and extends the lifespan of SMA animal models (Rodriguez-Muela et al., 2018), the direct contribution of autophagy to SMA pathology remains to be established.

## Acetylation

Lysine acetylation is a major PTM acting on many aspects of cellular metabolism (Choudhary et al., 2014). Co-activators CREB-binding protein (CBP) and its paralog E1A-binding protein (p300) have been reported to interact with SMN (Lafarga et al., 2018). CBP/p300 play central roles in gene expression regulation *via* various mechanisms, among which is the acetylation of histones to modulate chromatin conformation (Wang et al., 2013). Lysine 119 (K119) in SMN is predicted to be acetylated by *in silico* tools and has been validated by functional assays (Lafarga et al., 2018; Figure 1 and Table 1). Interestingly, K119 is also predicted to be sumoylated (Tapia et al., 2014) and could represent an opportunity for crosstalk between different SMN’s PTMs (see below).

K119 is located in the Tudor domain, a key portion of SMN responsible for interaction with several proteins residing in CBs, including Sm proteins and coilin. Consistently, mutation of K119 causes the formation of microbodies in the nucleus that are lacking the classical marks of the CBs, such as coilin, while retaining some characteristics of promyelocytic leukemia (PML) bodies (Lafarga et al., 2018). At the same time, loss of acetylation of SMN promotes the interaction with a different set of proteins, compared to wildtype SMN, causing impaired snRNP delivery to the spliceosome and splicing defects. Impaired assembly of the CBs following loss of acetylation at K119 is also mediated by a reduction of the nucleocytoplasmic transport of SMN through a reduction of diffusion of SMN (Figure 2). Finally, acetylation was shown to increase the stability of SMN by reducing the turnover of the protein (Lafarga et al., 2018).

Interestingly, histone deacetylase (HDAC) inhibitors, such as valproic acid and Trichostatin A, have been tested for the treatment of SMA, with the goal of increasing *SMN2* transcription levels by modulating promoter accessibility (Avila et al., 2007; Narver et al., 2008). In line with what has been summarized above, the lack of positive outcomes of these agents in clinical trials could be the result of a perturbation of other functions of acetylation of SMN that are lost upon treatment with HDAC inhibitors (Lafarga et al., 2018; Chaytow et al., 2021).

## Sumoylation

Small Ubiquitin-like Modifier (SUMO) conjugation (or sumoylation) has been increasingly proposed as a central mechanism for the regulation of multiple biological processes (Flotho and Melchior, 2013). SUMO proteins form covalent bonds with lysine residues on target proteins, a process mediated by activating (E1), conjugating (E2), and ligating (E3) enzymes (Pichler et al., 2017). Sumoylation is a highly dynamic process modulated by a family of SUMO-specific proteases that are responsible for SUMO deconjugation (Hickey et al., 2012). In addition to the covalent attachment of SUMO to its substrates, an increasing number of proteins, including SMN, have been shown to bind SUMO non-covalently *via* SUMO interaction motifs (SIMs; Tapia et al., 2014). Interactions between SUMO and SIM-containing proteins play an important role in nuclear bodies dynamics by mediating physical interactions between proteins (Jentsch and Psakhye, 2013).

Early work using immunocytochemistry and transfection experiments showed the presence of SUMO-1 and the SUMO E2 enzyme (UBC9) in a subset of CBs in undifferentiated neuron-like UR61 cells (Navascues et al., 2008). In addition, SUMO-1 was shown to transiently localize into CBs from adult nervous tissue in response to osmotic stress (Navascues et al., 2008). In a follow up study from the same group, SUMO1 has been reported to mainly localize in SMN-positive CBs, rather than the coilin-positive/SMN-negative ones (Tapia et al., 2014). A putative acceptor lysine (K119) was identified in the Tudor domain of SMN and SMN has been reported to be a SUMO1 substrate (Tapia et al., 2014; Figure 1). Interestingly, mutation of the acceptor lysine (K119R) did not abolish SMN sumoylation, suggesting that other acceptor lysines might be at play. Consistently, in addition to K119, lysines 55 and 285 have been predicted to be sumoylated (Figure 1 and Table 1). A combinatorial mutation of these residues might be necessary to determine which of the potential SUMO sites of SMN are functional.

Importantly, Tapia and colleagues reported a SUMO-interacting motif (SIM)-like sequence (V124/V125/V126) within the Tudor domain of SMN that mediates non-covalent interaction with SUMO (Tapia et al., 2014). While mutation of this SIM-like sequence does not affect covalent SMN sumoylation, the authors presented evidence that this domain is important for SMN interactions with SmD1 and coilin (Tapia et al., 2014). It is therefore possible that both covalent SUMO modification and non-covalent SUMO binding may regulate SMN function (Figure 2).

A recent work investigated the role of SUMO-SMN interactions using *in vitro* functional assays and two animal models of SMA (Riboldi et al., 2021). This study reported that, in addition to being modified by SUMO-1 and SUMO-2, SMN can interact non-covalently with SUMO through its SIM. A SIM-inactivated mutant of SMN (SMN-2VA) failed to assemble in high molecular weight complexes and showed a decreased activity in the assembly of spliceosomal snRNPs (Riboldi et al., 2021). In addition, SMN-2VA failed to localize in CBs, suggesting that a network of SUMO-SIM interactions contributes to the spatial organization of the SMN complex (Riboldi et al., 2021). Consistent with this view, Gemin3 and Gemin5 were reported to be sumoylated, with their sumoylation required for the interaction with SMN. Accordingly, global inhibition of sumoylation through knockdown of the E2 ligase UBC9 resulted in the mislocalization of SMN and other core components of the complex as well as altered the interaction of SMN with SUMO-modified components of the SMN complex (Figure 2).

Moreover, this study showed that sumoylation modulates the SMN activities that are relevant for SMA. Indeed, expression of SMN-2VA failed to prevent motor neuron development defects induced by SMN deficiency in zebrafish. In SMA mice, expression of SMN-2VA prevented motor neuron death and improved NMJ denervation, while failing to rescue sensory-motor connectivity deficits (Riboldi et al., 2021). Taken together, these findings establish sumoylation as an important determinant of the integrity and function of the SMN complex and link this PTM to select aspects of sensory-motor circuit dysfunction in animal models of SMA. Accordingly, a recent study reported that SMN can be modified by SUMO2 and can be desumoylated by the SUMO/sentrin-specific protease SENP2 (Zhang et al., 2021). SENP2 deficiency induced SMN hyper-sumoylation and promoted the degradation of SMN by the UPS pathway. Importantly, SENP2-deficient mice developed an SMA-like phenotype, suggesting a role for sumoylation in the etiology of the disease (Zhang et al., 2021).

## Crosstalk between PTMs of SMN

The many sites in SMN that undergo PTMs raise the question about the relative significance of a single-site modification in the regulation of SMN complex function. Indeed, in most cases, the contribution of a single site modification to the overall protein function is barely decisive. Instead, widespread crosstalk between modifications is at play and the integration of all this information determines a context-dependent functional output. In this context, crosstalk is intended as the combinatorial action of multiple PTMs on the same or on different proteins (Leutert et al., 2021). There are several ways in which PTM crosstalk can act and cooperative modification crosstalk where a modification promotes the effect of another or antagonistic modification crosstalk where a modification blocks the effect of another are the main modes of action (Hunter, 2007).

The simplest case for antagonistic modification crosstalk is that the same amino acid in SMN can be modified by different modification types (Figure 1). For instance, lysine 55 is predicted to be modified by both ubiquitin and SUMO, with a possible competition between corresponding modifying enzymes to access this site. In addition, lysine 119 has been reported to be a target for both acetylation and sumoylation. However, functional experiments with mutant K119R showed reduced levels of acetylation of SMN, but not of SUMO1 modification (Lafarga et al., 2018). While SMN has been reported to be modified mostly by SUMO2 (Riboldi et al., 2021; Zhang et al., 2021), the contribution of K119 to this modification has not been assessed. Interestingly, Zhang et al. (2021) reported that SUMO2 hyper-modification of SMN leads to an increase in its level of acetylation and ubiquitination with consequent reduction in protein stability.

An opportunity for cooperative PTM crosstalk comes from the PKA-mediated phosphorylation at non-canonical sites of SMN which has been reported to promote SMN stability by modulating its interaction with Gemins and facilitating its incorporation into high molecular weight complexes (Burnett et al., 2009; Wu et al., 2011). In addition, SUMO-SIM interactions can be facilitated by the phosphorylation of residues adjacent to the SIM hydrophobic core (Yau et al., 2021). In this context, it is interesting to note that tyrosine 127 is located within SMN’s SIM and its phosphorylation could provide additional negative charges for favorable electrostatic interaction with SUMO. Interestingly, loss of Y127 phosphorylation has been shown to abolish SMN accumulation in CBs (Husedzinovic et al., 2014). However, whether Y127 phosphorylation facilitates SUMO-SIM interactions in the context of the SMN complex has not been determined yet.

## Conclusions and future perspectives

Despite extensive results about multiple modification types and the enzymes involved in the process, our understanding of the role of these PTMs in SMN complex functions and their contribution to SMA pathology is still in its infancy. It is currently unclear whether PTMs of SMN are triggered by cellular stresses and whether are integrated into regulatory networks as well as whether specific modification states can help SMN differentiate among the myriad of interacting partners (Figure 2). Evidence in this direction is emerging for a network of SUMO-SIM interactions within the SMN complex that likely contributes to the integrity and spatial organization of the complex (Riboldi et al., 2021). In this context, SMN has been reported to interact with two RBPs linked to ALS, such as TDP-43 and FUS (Yamazaki et al., 2012; Tsuiji et al., 2013). Interestingly, TDP-43 has been found to be sumoylated while FUS might work as a SUMO E3 ligase (Oh et al., 2010; Maraschi et al., 2021). Therefore, it is tempting to speculate that sumoylation can influence the interaction of SMN with TDP-43 and FUS. In addition, it is not known whether PTMs of SMN can be regulated at the tissue and cellular level to finetune SMN functions in different cell types. Finally, although crosstalk between SMN’s modifications has been reported, how the combinatorial interplay of different PTMs and interaction partners is coordinated and its contribution to SMN function is still unknown.

Liquid phase separation is an emerging concept to conceptualize a novel mode of protein interaction (Hnisz et al., 2017). Recently, a role for phosphorylation in the regulation of SMN phase separation has been proposed and could serve as a paradigm for further elucidation of the role of SMN’s PTMs in the assembly of subnuclear structures such as Gems and CBs (Schilling et al., 2021). In this context, interaction between SMN’s Tudor domain and DMA-modified proteins has been proposed to define the specific composition of certain condensate membrane-less organelles such as Gems and CBs (Courchaine et al., 2021). Interestingly, a network of SUMO-SIM interactions within the SMN complex has also been reported to regulate the formation of Gems and CBs (Riboldi et al., 2021). However, the crosstalk between these different modifications of SMN in the assembly of these subnuclear structures has not been investigated yet.

Despite the huge success of SMA therapies (Mercuri et al., 2022), a significant number of patients do not, or respond less well to these SMN augmentation approaches. Therefore, there is still potential for improvement provided by combinatorial therapeutic strategies addressing molecular networks which are no longer responsive to SMN restoration (Hensel et al., 2020). In this context, the identification of enzymes depositing and modulating SMN’s PTMs could inspire new ideas for effective therapeutic options that work together with established strategies to reduce disease burden in SMA patients. A small percentage of patients with SMA present point mutations on the SMN gene, usually in trans with the more classical deletion of the *SMN1* gene. It is plausible that amino acid changes at posttranslationally-modified sites can have a direct contribution to SMA pathogenesis. In this context, a total of 12 patient mutations have been identified in confirmed or putative phospho-sites, however their impact on the SMA phenotypes has not been determined (Detering et al., 2022). Therefore, a deeper understanding of the role of PTMs in SMN biology will be important to fully dissect the multifaceted functions of SMN and their link to SMA pathology and ultimately broaden the knowledge necessary for the development of increasingly effective therapies for SMA.

## Author contributions

GR, IF, PR, and FL: contributed to the writing and editing of the manuscript. All authors contributed to the article and approved the submitted version.
